# Microglial cGAS drives neuroinflammation in the MPTP mouse models of Parkinson's disease

**DOI:** 10.1111/cns.14157

**Published:** 2023-03-13

**Authors:** Chunmei Ma, Ying Liu, Sheng Li, Chanyuan Ma, Jiajia Huang, Shuang Wen, Shuo Yang, Bingwei Wang

**Affiliations:** ^1^ Department of Immunology, State Key Laboratory of Reproductive Medicine, Jiangsu Key Lab of Cancer Biomarkers, Prevention and Treatment, Collaborative Innovation Center for Personalized Cancer Medicine, Gusu School Nanjing Medical University Nanjing China; ^2^ Department of Pharmacology Nanjing University of Chinese Medicine Nanjing China; ^3^ Department of Laboratory Medicine, Huashan Hospital, Shanghai Medical College Fudan University Shanghai China

**Keywords:** antiviral inflammatory signaling, cGAS, MPTP, neuroinflammation, Parkinson's disease

## Abstract

**Background:**

Neuroinflammation has been widely accepted as a cause of the degenerative process. Increasing interest has been devoted to developing intervening therapeutics for preventing neuroinflammation in Parkinson's disease (PD). It is well known that virus infections, including DNA viruses, are associated with an increased risk of PD. In addition, damaged or dying dopaminergic neurons can release dsDNA during PD progression. However, the role of cGAS, a cytosolic dsDNA sensor, in PD progression remains unclear.

**Methods:**

Adult male wild‐type mice and age‐matched male cGAS knockout (*cGas* ^−/−^) mice were treated with MPTP to induce neurotoxic PD model, and then behavioral tests, immunohistochemistry, and ELISA were conducted to compare disease phenotype. Chimeric mice were reconstituted to explore the effects of cGAS deficiency in peripheral immune cells or CNS resident cells on MPTP‐induced toxicity. RNA sequencing was used to dissect the mechanistic role of microglial cGAS in MPTP‐induced toxicity. cGAS inhibitor administration was conducted to study whether GAS may serve as a therapeutic target.

**Results:**

We observed that the cGAS‐STING pathway was activated during neuroinflammation in MPTP mouse models of PD. cGAS deficiency in microglia, but not peripheral immune cells, controlled neuroinflammation and neurotoxicity induced by MPTP. Mechanistically, microglial cGAS ablation alleviated the neuronal dysfunction and inflammatory response in astrocytes and microglia by inhibiting antiviral inflammatory signaling. Additionally, the administration of cGAS inhibitors conferred the mice neuroprotection during MPTP exposure.

**Conclusions:**

Collectively, these findings demonstrate microglial cGAS promote neuroinflammation and neurodegeneration during the progression of MPTP‐induced PD mouse models and suggest cGAS may serve as a therapeutic target for PD patients.

**Limitations of the Study:**

Although we demonstrated that cGAS promotes the progression of MPTP‐induced PD, this study has limitations. We identified that cGAS in microglia accelerate disease progression of PD by using bone marrow chimeric experiments and analyzing cGAS expression in CNS cells, but evidence would be more straightforward if conditional knockout mice were used. This study contributed to the knowledge of the role of the cGAS pathway in PD pathogenesis; nevertheless, trying more PD animal models in the future will help us to understand the disease progression deeper and explore possible treatments.

## Graphical abstract

Model for microglial cGAS mediates neuron protection against MPTP. During MPTP treatment, microglial cGAS‐STING pathway is activated by the damaged dsDNA released from dying DA neuron, and then promoting translocation of IRF3 and NF‐κB to the nucleus to activate transcription of antiviral inflammatory cytokines. The antiviral inflammatory cytokines exacerbate neuroinflammation and neurodegeneration by promoting neuronal death and phenotypic switch in astrocytes and microglia from a homeostatic to an inflammatory state.

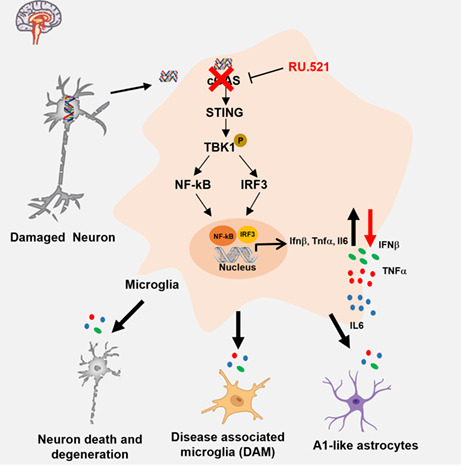

## 1 BACKGROUND

Parkinson's disease (PD), a multifactorial and age‐related neurodegenerative disease, is characterized by the dramatic loss of dopaminergic neurons in the substantia nigra (SN). PD causes motor symptoms with bradykinesia, resting tremors, rigidity, and many non‐motor disturbances.^1^ It is well known that inflammatory attacks can induce disability and loss of dopaminergic neurons, thereby contributing to the pathogenesis of PD.^2^ Notably, virus infections, including Epstein–Barr virus, herpes simplex virus, hepatitis C virus, and Coronavirus disease‐19 were associated with an increased risk of PD,^3^, ^4^, ^5^, ^6^ suggesting virus infection‐associated inflammation may be a probable contributor to the pathogenesis of PD. Since several epidemiological studies reported that non‐steroidal anti‐inflammatory drugs (NSAID) reduce the risk of PD,^7^, ^8^, ^9^ much attention has been devoted to understanding the benefits of targeting the inflammatory response to functional recovery after PD onset.

More precise anti‐inflammatory targets and therapeutic strategies must be further explored. During PD, apoptotic loss of neurons and dsDNA breaks occur in the brain. However, the role of broken genomic dsDNA and the mechanisms through which these dsDNA regulate PD neuroinflammation and pathogenesis remains largely unknown. Therefore, exploring DNA sensor‐mediated antiviral inflammation may provide a new potential target for the treatment of PD.

Several reports have shown the role of innate immune cells and inflammation in PD pathogenesis.^10^, ^11^, ^12^ Innate immune cells, including macrophages, monocytes, dendritic cells, and microglia (the specialized population of macrophages‐like cells in the brain), are essential in triggering an inflammatory response to infection or sterile injury by their pattern recognition receptors (PRRs).^13^, ^14^ Among the PRRs, the cyclic GMP‐AMP synthase (cGAS) is a primary cytosolic double‐stranded DNA sensor that initiates inflammation in response to infection or sterile tissue damage.^15^ Upon DNA binding, cGAS is activated to catalyze the synthesis of 2′3′‐cGAMP, which binds and activates the adaptor stimulator of interferon genes (STING).^16^ STING, in turn, activates TBK1 and IKK, leading to the activation of the transcription factors IRF3 and NF‐κB, respectively, which induce the expression of type‐I IFNs and other immune regulatory molecules.^17^ cGAS has been reported to play essential roles in host defense, tumor growth, and inflammatory diseases.^15^ It is worth noting that the cGAS pathway's effect on the central nervous system (CNS) has begun to unravel. Recently, cGAS has been reported to be involved in the pathogenesis of some neurodegenerative progress, such as Huntington's disease (HD) and experimental autoimmune encephalomyelitis (EAE).^18^, ^19^ However, the exact function of cGAS in the context of PD is largely unknown.

In the present study, to explore the exact role of cGAS in the progression of PD, we utilized MPTP‐induce neurotoxic PD model and bone marrow chimeric experiment. We found that microglial cGAS exacerbates pathologic neuroinflammation and neurotoxicity by positively regulating antiviral inflammatory signaling during MPTP exposure, establishing a novel potential target for PD therapeutics.

## 2 MATERIALS AND METHODS

### 2.1 Animals

Male mice with the C57BL/6 background were used in this study. *cGas* ^−/−^ mice were purchased from The Jackson Laboratory (JAX stock #026554). The progeny of *cGas* ^+/−^ intercrosses were genotyped by PCR analysis of DNA isolated from the tail using the following three primers: 5′‐ATA TTT CCCCCTGTGTTG GA‐3′, 5′‐GTG CCAGGTGACACAACATC‐3′, 5′‐CGGATGGATGAACAAACAGA‐3′. The band size of wild type and mutant is 188 and 298 bp, respectively. The knockout mice bred, grew and developed similarly to WT mice, same as previously reported.^20^ All mice were housed under pathogen‐free conditions and on a 12 h light/dark cycle at 25°C with standard food and water available ad libitum. Male mice between 8 and 10 weeks of age were assessed in the MPTP model. All animal procedures were conducted according to the US National Institutes of Health Guide for the Care and Use of Laboratory Animals and were approved by the Ethical Review Committee for Laboratory Animal Welfare of Nanjing Medical University and Nanjing University of Chinese medicine.

### 2.2 In vivo experimental treatments

The mice received intraperitoneal injection of MPTP at a dose of 20 mg/kg four times with 2 h intervals between injections. Seven days later, the mice underwent behavioral testing, and then brain samples were collected for histological analyses, quantitative polymerase chain reaction (qPCR), and western blotting. In some case, mice received MPTP/p administration as described previously.^21^ In brief, 10 doses of MPTP HCL in saline plus probenecid in dimethyl sulfoxide were given twice a week for 5 weeks. Each time, the mice were subcutaneously injected with MPTP‐HCl (20 mg/kg), and 1 h later, the mice were intraperitoneally injected with probenecid (250 mg/kg, Macklin, P822732). Animals underwent behavioral testing or were sacrificed for further analysis 7 days after the last treatment. For cGAS inhibitor RU.521 (MCE, HY‐114180) treatment, the mice were intraperitoneally injected with RU.521 (10 mg/kg) daily starting the same day as MPTP administration.

### 2.3 Bone marrow chimeras

Recipient *cGas* ^−/−^ and WT mice were irradiated at a lethal dose of 10 Gy to kill the bone marrow cells. At 24 h post‐irradiation, the recipients were intravenously injected with bone marrow cells (1 × 10^7^) from donor *cGas* ^−/−^ or WT mice. Two months after bone marrow transplantation, the chimeric mice were treated with the MPTP.

### 2.4 Open field test

As previously described, an open field test was conducted before all other behavioral tests were conducted.^22^ The open field, made of white plastic, consisted of a square arena of 50 cm × 50 cm. The square arena was divided into a peripheral and a central area, and each sub‐square occupied 50% of the total open area. Before the start of the test, the mice were brought to the room to adapt to the environment for 1 h, and then mice were placed into the open field device with low light for 15 min. During the test, the total traveled distance, movement speed, and central area duration were automatically recorded.

### 2.5 Pole test

As previously described, a pole test was used to evaluate mouse balance and coordination.^23^ Briefly, the mice were placed on a vertical pole with their head up (the pole was 0.5 m long and 1 cm in diameter). The total time from the top to descend back into the cage was recorded. Before the start of the test, the training experiment was repeated five times continuously, with 30 min intervals between each training trial. The following day after the training experiment, each mouse was tested thrice with 3 min intervals between tests. The average value of the three tests was recorded.

### 2.6 Accelerating rotarod test

The accelerating rotarod test was conducted as described previously.^22^ The mice were acclimated to the rotarod apparatus for training, which accelerated from 5 to 30 rpm in 300 s. Each mouse was trained for 3 consecutive days with five trials per day with 30 min intervals between each trial. On the day of the experiment, three trials were conducted with 3 min intervals between each test. Latency to fall was recorded, and the average value of the three trials of mice was finally calculated. After each test, the excrements were cleaned, and the arena was decontaminated with 75% alcohol.

### 2.7 Immunohistochemistry and immunofluorescence staining

After all behavioral tests, mice were anesthetized and perfused with PBS and 4% paraformaldehyde (PFA) to collect brain samples. For frozen sections, brains were fixed in 4% PFA for 48 h and put into 30% sucrose solution for dehydration and then embedded in OCT. The 25 μm thickness slices were prepared for further analysis. For immunohistochemistry staining, tissue sections or primary neurons were deparaffinized by 3% H_2_O_2_ for 10 min and blocked with 5% goat serum for 60 min at room temperature. The sections or neurons cells were then incubated with a primary antibody reactive with Tyrosine hydroxylase (TH, 1:500, AB152, Merck‐Millipore) at 4°C for 12 h, followed by incubation with biotinylated goat anti‐rabbit IgG secondary antibody (1:300, BA1003, Boster) for 1 h at 37°C, and incubated with horseradish peroxidase (HRP)‐labeled goat anti‐rabbit (1:300, 31460, Thermo Fisher Scientific) for 1 h at 37°C. The number of TH‐positive cells was determined by stereological methods, as previously described.^24^ In briefly, TH‐ stained neurons were counted in the right and left SNpc of every five sections throughout the entire extent of the SNpc. And to avoid double counting of neurons with unusual shapes, TH‐ stained cells were counted only when their nuclei were optimally visualized, which occurred only in one focal plane. For immunofluorescence staining, tissue sections of primary microglia and astrocyte were blocked with 5% goat serum for 60 min at room temperature and then incubated with primary antibodies reactive with ionized calcium‐binding adaptor molecule 1 (IBA1, 1:500, 019‐19741, Wako), glial fibrillary acidic protein (GFAP, 1:500, G3893, Sigma‐Aldrich), γH2AX (1:100, ab11174, Abcam), Complement C3 (1:200, PA5‐21349, ThermoFisher), apolipoprotein E (APOE, 1:100, ab1906, Abcam), TH or p‐TBK1 (1:1000, 5483s, Cell Signaling Technology) at 4°C for 12 h. The sections or primary cells were then incubated with fluorescent‐labeled goat anti‐rabbit (1:500, 111‐545‐144, Jackson ImmunoResearch) and goat anti‐mouse (1:500, 115‐165‐075, Jackson ImmunoResearch). Fluorescence intensities were analyzed by Image Pro Plus software.

### 2.8 Western blot analysis

Brain SN tissue was prepared as single‐cell suspensions previously described.^25^ In brief, brain SN tissue was digested with DNase I (10 U/mL, Roche) and collagenase IV (0.5 mg/mL, Sigma‐Aldrich) in RPMI 1640 at 37°C for 60 min. Single‐cell suspensions were filtered through a 70‐μm cell strainer and then centrifuged through a 30% Percoll density gradient (GE Healthcare). Subsequently, the cells were collected from the bottom of the tube to lyse in NP‐40 lysis buffer containing protease inhibitor cocktail. The protein lysates were centrifuged at 12,000 *g* for 10 min, and protein concentrations were determined by PierceMT B.C.A. protein assay. Samples were resolved by SDS‐PAGE, transferred to nitrocellulose membranes, and reacted with appropriate antibodies. Antibodies reactive with cGAS (1:1000, 31659s), interferon regulatory factor 3 (IRF3, 1:1000, 4302s), TANK binding kinase 1 (TBK1, 1:3000, 3013s), p‐TBK1 (1:1000, 5483s), and stimulator of interferon genes (STING, 1:1000, 13647s) were purchased from Cell Signaling Technology. β‐actin (1:5000, A1978) was purchased from Sigma‐Aldrich, IRDye 680RD anti‐mouse (1:5000, 926‐68070) and IRDye 800CW anti‐rabbit (1:5000, 926‐32211) were purchased from LI‐COR Biosciences, and anti‐goat‐HRP (1:3000, 31402) were purchased from Thermo Fisher Scientific. Immunoreactivity was visualized with an Odyssey Imaging System (LI‐COR Biosciences) or by enhanced chemiluminescence.

### 2.9 Quantitative polymerase chain reaction (qPCR) analysis

Total RNA was extracted using TRIzol reagent (Invitrogen) following the manufacturer's instructions. The cDNAs were synthesized using a cDNA synthesis kit (Vazyme) according to the manufacturer's protocols. Quantitative PCR was performed using SYBR Green Supermix (Vazyme). The following primers were used:

**Table:** 

	F (5′ → 3′)	R (5′ → 3′)
*Hprt*	GTCCCAGCGTCGTGATTAGC	TGGCCTCCCATCTCCTTCA
*cGas*	GAGGCGCGGAAAGTCGTAA	TTGTCCGGTTCCTTCCTGGA
*Sting*	GGTCACCGCTCCAAATATGTAG	CAGTAGTCCAAGTTCGTGCGA
*Ifit1*	CTGAGATGTCACTTCACATGGAA	GTGCATCCCCAATGGGTTCT
*Tnfα*	CCCTCACACTCAGATCATCTTCT	GCTACGACGTGGGCTACAG
*Ifnβ*	CAGCTCCAAGAAAGGACGAAC	GGCAGTGTAACTCTTCTGCAT
*Irf7*	GAGACTGGCTATTGGGGGAG	GACCGAAATGCTTCCAGGG
*Irf3*	GAGAGCCGAACGAGGTTCAG	CTTCCAGGTTGACACGTCCG
*Isg15*	GGTGTCCGTGACTAACTCCAT	TGGAAAGGGTAAGACCGTCCT
*Cxcl10*	AAGTGCTGCCGTCATTTTCTG	TTCCCTATGGCCCTCATTCTC
*Usp18*	TTGGGCTCCTGAGGAAACC	CGATGTTGTGTAAACCAACCAGA
*Ifit3*	CCTACATAAAGCACCTAGATGGC	ATGTGATAGTAGATCCAGGCGT
*Ifi44*	AACTGACTGCTCGCAATAATGT	GTAACACAGCAATGCCTCTTGT
*Oasl2*	TTTGCGGAGGATCAGGTACT	TGATGGTGTCGCAGTCTTTGA
*Apoe*	CTGACAGGATGCCTAGCCG	CGCAGGTAATCCCAGAAGC
*Il6*	CTTGGGACTGATGCTGGTGAC	GCCATTGCACAACTCTTTTCTC
*C3*	CCAGCTCCCCATTAGCTCTG	GCACTTGCCTCTTTAGGAAGTC
*S100β*	TGGTTGCCCTCATTGATGTCT	CCCATCCCCATCTTCGTCC

### 2.10 Enzyme‐linked immunosorbent assay (ELISA)

Primary microglia were stimulated with cell‐free dsDNA from the MPP^+^‐treated SH‐SY5Y neuronal cell line, and then the CM were collected and stored at −80°C for the ELISA assay. Brain SN tissues were dissected and homogenized in lysis buffer, and then supernatants were collected for the ELISA assay. The medium, as mentioned earlier, and supernatants were measured for IFNβ (424001), CXCL10 (DY466), and TNFα (DY410) according to ELISA kit instructions (Invitrogen, R&D Systems).

### 2.11 Flow cytometry

Brain SN tissue was prepared as single‐cell suspensions previously described.^25^ In brief, brain SN tissue was digested with DNase I (10 U/mL, Roche) and collagenase IV (0.5 mg/mL, Sigma‐Aldrich) in RPMI 1640 at 37°C for 60 min. Cells were filtered through a 70 μm cell strainer and centrifuged through a 30% Percoll density gradient. After intensive washing, single‐cell suspensions were stained with FVD eFluor 506, anti‐CD45 (11‐045182, Invitrogen), anti‐CD11b (17‐0112‐82, eBioscience), and anti‐P2RY12 (848003, BioLegend) for FACS analysis. For p‐TBK1 staining, cells were fixed and permeabilized with the Intracellular Fixation & Permeabilization Buffer Set (00‐8333‐56, eBioscience) and then subjected to incubate with primary p‐TBK1 antibody for 1 h. Subsequently, cells were then incubated with Alexa Fluor 488 for 1 h. All flow cytometry was performed on an Attune NxT flow cytometer (Thermo Fisher Scientific), and data were analyzed by FlowJo 10.0.7 software.

### 2.12 RNA‐seq analysis

For RNA‐seq, CD45^+^CD11b^+^P2RY12^+^ microglia were sorted from mice of MPTP induction on a BD FACS Aria. Total RNA isolation was extracted with a Trizol reagent. cDNA library construction and RNA‐seq were performed by LC‐Bio Technology Co. Ltd. with Illumina Novaseq6000. Clean reads were mapped to the mouse genome by HISAT2. Calculated the matched reads and then normalized them to FPKM. Calculated the fold change for all possible comparisons, and a 1.1‐fold cut‐off was used to select genes with expression changes. KEGG pathway and GO‐BP analysis were performed using the R package, using significantly differentially expressed genes as target genes. Raw data and processed files have been deposited in the NCBI Sequence Read Archive (SRA) database under the accession code PRJNA904829.

### 2.13 Primary microglial culture and conditioned medium collection

Meninges and blood vessels were carefully stripped from the cerebral cortices of newborn mice at 1–3 days old. After dissection, the cerebral cortices were collected to digest with 0.25% trypsin–EDTA, followed by a wash in Hank's balanced salt solution (HBSS) containing 10% fetal bovine serum (FBS). The cell suspensions were passed through a cell strainer (70 μm). The obtained single‐cell suspension was seeded into poly‐D‐lysine (PDL, Sigma)–pre‐coated flasks and cultured in Dulbecco's modified Eagle's medium (DMEM)/F12 supplemented with 10% FBS at 37°C and 5% CO_2_. The culture medium was changed every 3 days for 14 days. The microglia were collected by shaking the flasks gently and cultured in plates pre‐coated with PDL overnight. The next day, cell‐free dsDNA released from SH‐SY5Y neuronal cells (pretreated with 1‐methyl‐4‐phenylpyridinium, MPP^+^) was collected with a MagMAX Cell‐Free DNA Isolation Kit (A29319, Thermofisher). The dsDNA was transfected into microglia (2 μg/10^6^ cells) with lipofectamine 2000 (2 μL/mL). After 8 h, the conditioned medium (CM) from microglia was collected for measurement of cytokines by ELISA or filtered using 0.22 μm pore filters and stored at −80°C until use.

### 2.14 Primary astrocytes culture and treatment

Primary cultures of hippocampus astrocytes were prepared from neonatal mice, as described previously.^26^ After 7 days, astrocytes were purified using more than four repetitions of trypsinization and re‐plating. Astrocytes were seeded in plates pre‐coated with PDL overnight, then incubated with CM (CM:DMEM/F12 = 1:2) for 24 h. Then cells were collected to measure cytokine gene expression by qPCR and immunofluorescence staining.

### 2.15 Primary neurons culture and treatment

Primary neurons were prepared from the ventral mesencephalon of fetuses (E15–16) by treatment with 0.125% trypsin EDTA, as described previously.^27^ The neurons were cultured in a neurobasal medium supplemented with 2% B27 and 0.5 mM glutamine for 6 days and treated with CM (CM:neurobasal = 1:2) for 24 h.

### 2.16 Hoechst staining

Neurons were fixed with 4% PFA for 30 min and stained with Hoechst 33324 (1:1000 dilution) for 10 min. Apoptotic neurons were quantified by imaging in a fluorescence microscope (Olympus BX 60).

### 2.17 Statistical analysis

Statistical analysis was performed with GraphPad Prism 8.0 software. The normality of the data was analyzed using the Shapiro–Wilk test. For data with normal distribution, comparisons were performed using the unpaired *t*‐test or multiple *t*‐tests. Continuous variables data with non‐normal distributions were analyzed by the Mann–Whitney test. Data are expressed as the means ± standard error of the mean (SEM).

## 3 RESULTS

### 3.1 The cGAS‐STING pathway is involved in the pathogenesis of PD

**FIGURE 1:**
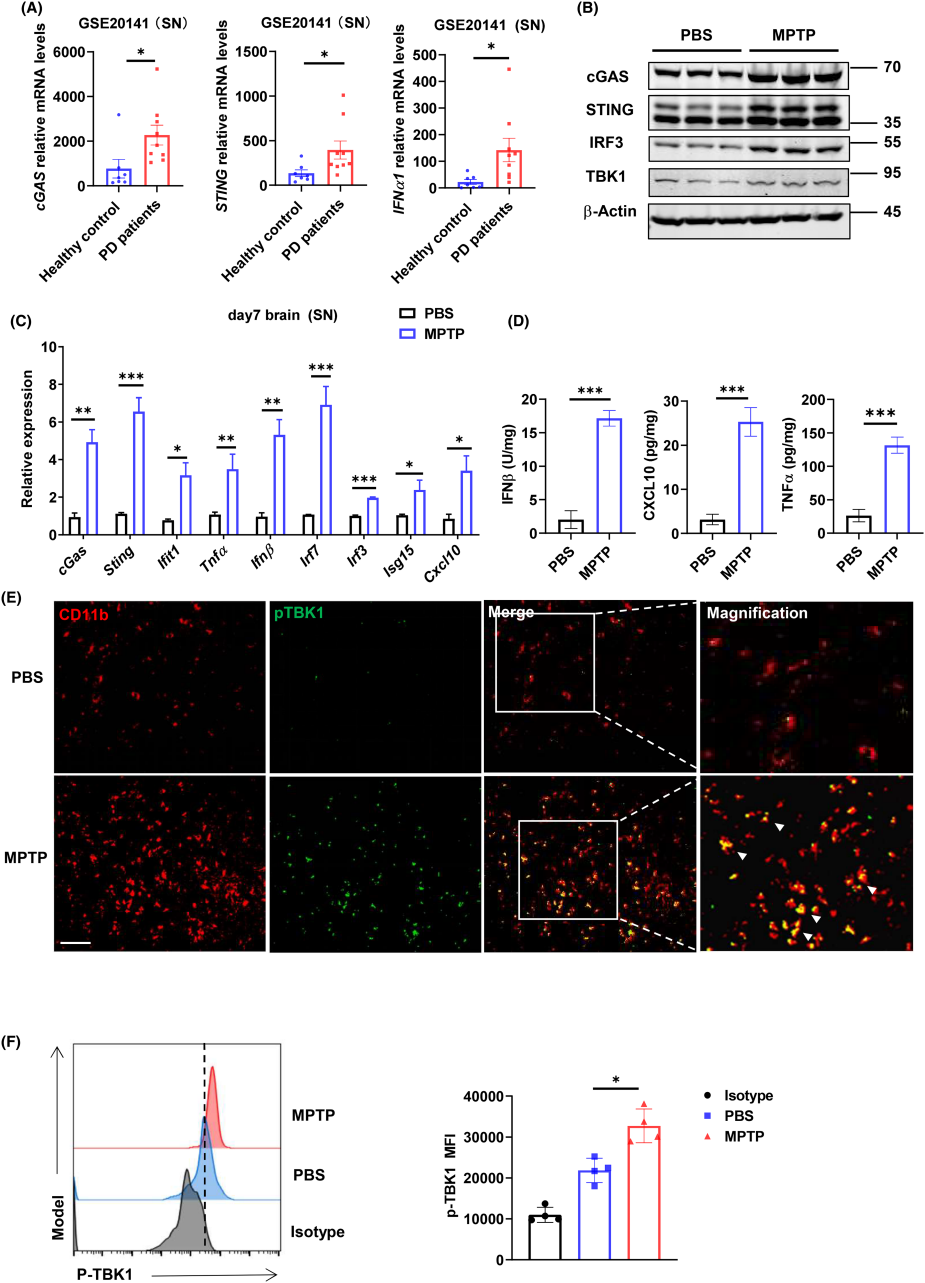
cGAS‐STING pathway is activated during the pathogenesis of PD. (A) Comparison of cGAS, STING and IFNα1 transcript in SN tissue between healthy controls and PD patients from public datasets in GEO (healthy controls = 7/group, PD patients = 9/group). (B) Western blot analysis of cGAS, STING, IRF3, TBK1, and β‐Actin (loading control) in the brain SN tissue of the indicated mice (*n* = 3/group). (C) Quantitative PCR analysis of indicated genes in the brain SN tissue of PBS or MPTP‐induced mice (*n* = 4/group). Data were normalized to the reference gene, *Hprt*. (D) ELISA assay of IFNβ, CXCL10 and TNFα in the brain SN tissue of PBS or MPTP‐induced mice (*n* = 4/group). (E) Representative immunofluorescence staining for p‐TBK1 (green) in microglia (CD11b, red) of midbrain tissues from PBS or MPTP‐induced mice (*n* = 4/group). The merge of p‐TBK1with CD11b is indicated by white arrowheads. Scale bar, 50 μm. (F) Flow cytometry analysis of p‐TBK1 in the brain SN tissue of PBS or MPTP‐ induced mice (*n* = 4/group). Mean fluorescence intensity (MFI) are detailed with representative overlaid histograms (left) and quantified summary graph (right). Data are representative of two independent experiments for (B). Data are pooled from three independent experiments for (C–F). Error bars show means ± SEM. Unpaired *t*‐test for (A) and (F). Multiple *t*‐test for (C) and (D).

Public datasets in GEO showed that *cGAS*, *STING*, and *IFNα1* expression increased in the SN tissues of PD patients compared with healthy donors (Figure 1A), suggesting that the cGAS‐STING pathway may be involved in regulating the progression of PD.^28^ To further explore the cGAS‐STING activation, we constructed the MPTP‐induced neurotoxic PD model. In brief, MPTP was intraperitoneally injected into mice at a dose of 20 mg/kg four times at 2 h intervals. Then, behavioral testing and brain tissue isolation were performed 7 days later (Figure S1A). QPCR and western blot analysis revealed substantially increased expressions of cGAS and STING in the SN area of MPTP mice (Figure 1B,C). We also observed significantly increased expressions of cGAS‐STING pathway‐related molecules, such as IRF3, TBK1, IFNβ, CXCL10, and TNFα in the SN area of MPTP mice (Figure 1B–D). Accordingly, FACS and immunofluorescence analysis showed an increase in the phosphorylation of TBK1 (Figure 1E,F), suggesting the cGAS‐STING pathway was activated in the MPTP model. Collectively, these data suggest that cGAS‐STING activation is associated with PD progression.

### 3.2 cGAS deficiency protects against MPTP toxicity

**FIGURE 2:**
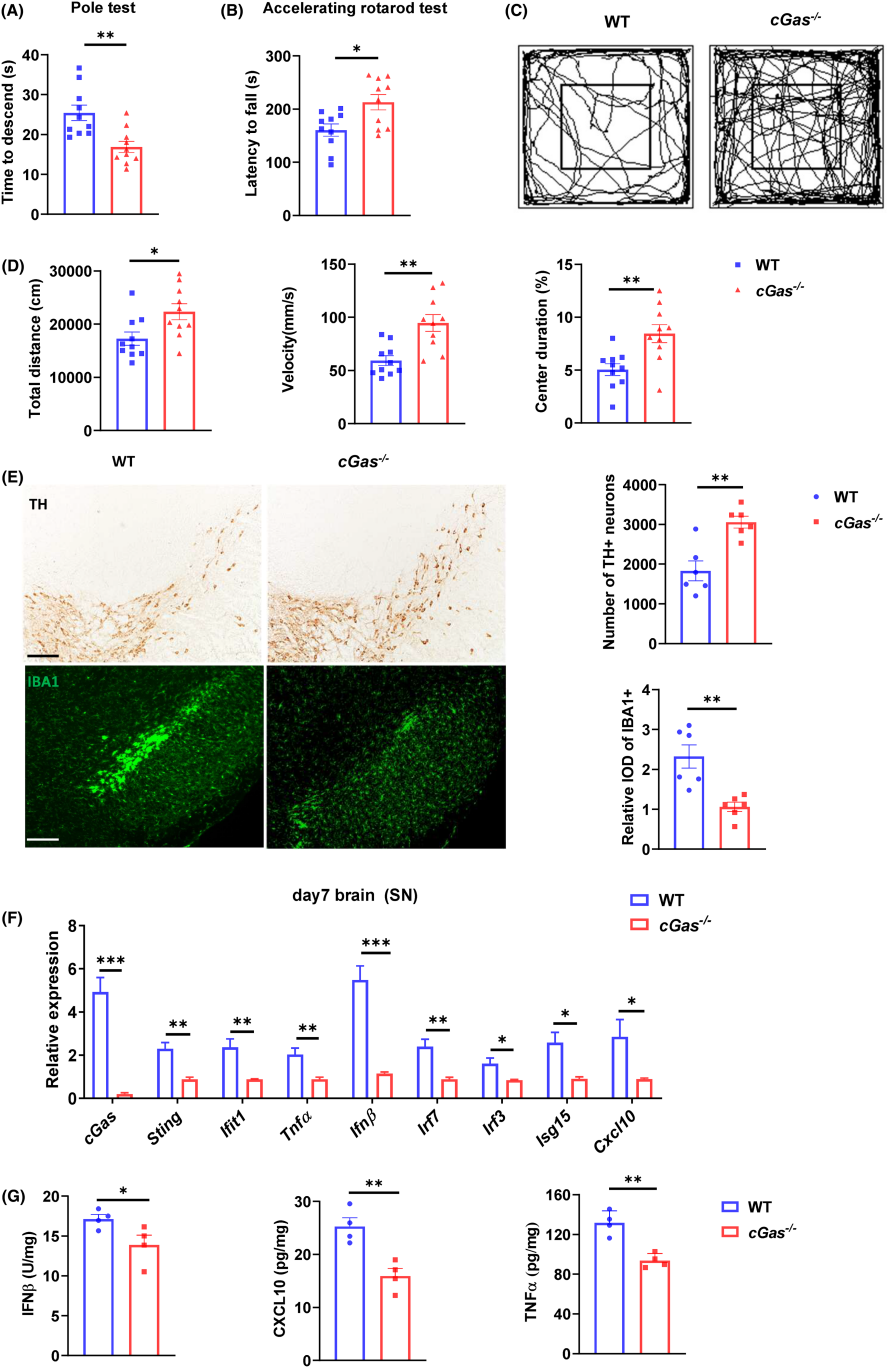
cGAS deficiency protects mice from MPTP toxicity. (A) Time to descend during pole test analysis of MPTP‐treated WT or *cGas* ^−/−^ mice (*n* = 10/group). (B) Latency to fall during the accelerating rotarod test analysis of MPTP‐induced WT or *cGas* ^−/−^ mice (*n* = 10/group). (C and D) Open field test analysis of MPTP‐induced WT or *cGas* ^−/−^ mice (*n* = 10/group). Data are presented as representative movement tracks in (C), quantified total traveled distance, movement speed and percentage of center area duration in (D) (*n* = 10/group). (E) Immunohistochemistry and immunofluorescence analysis of TH neurons and microglia (IBA1, green) in the brain SN tissue from indicated mice (*n* = 6/group). Data are presented as representative pictures (left), quantified number of TH neurons and relative IOD of IBA1 (right). Scale bar, 200 μm. (F) Quantitative PCR analysis of indicated genes in the brain SN tissue of PBS or MPTP‐induced mice (*n* = 4/group). Data were normalized to the reference gene, *Hprt*. (G) ELISA analysis of IFNβ, CXCL10 and TNFα in the brain SN tissue of PBS or MPTP‐induced mice (*n* = 4/group). Data are pooled from three independent experiments. Error bars show means ± SEM. Multiple *t*‐test.

To directly elucidate whether cGAS is involved in limiting or preventing MPTP‐induced toxicity, we treated age‐matched cGAS knockout (*cGas* ^−/−^) and wild‐type (WT) mice with MPTP to compare their phenotype (Figure S1A), and the ablation of *cGas* was demonstrated using western blot (Figure S1B). The pole and accelerating rotarod test showed *cGas* ^−/−^ mice descend faster and fall less easily than WT mice (Figure 2A,B). The open test showed that *cGas* ^−/−^ mice traveled more distance and spent more time in the center (Figure 2C,D). Consistently, *cGas* ^−/−^ mice showed less loss of TH‐positive neurons and decreased the number of activated microglial cells and GFAP^+^ astrocytes in both SN and dorsal striatum area (Figure 2E, Figure S1C). Additionally, we found lower levels in many factors associated with cGAS‐dependent inflammation, including *Ifit1*, *Tnfα*, *Ifnβ*, *Irf7*, *Irf3*, *Isg15*, and *Cxcl10* in the SN area of *cGas* ^−/−^ mice (Figure 2F,G). Thus, cGAS promotes neuroinflammation and exacerbates neurotoxicity elicited by MPTP treatment.

**FIGURE 3:**
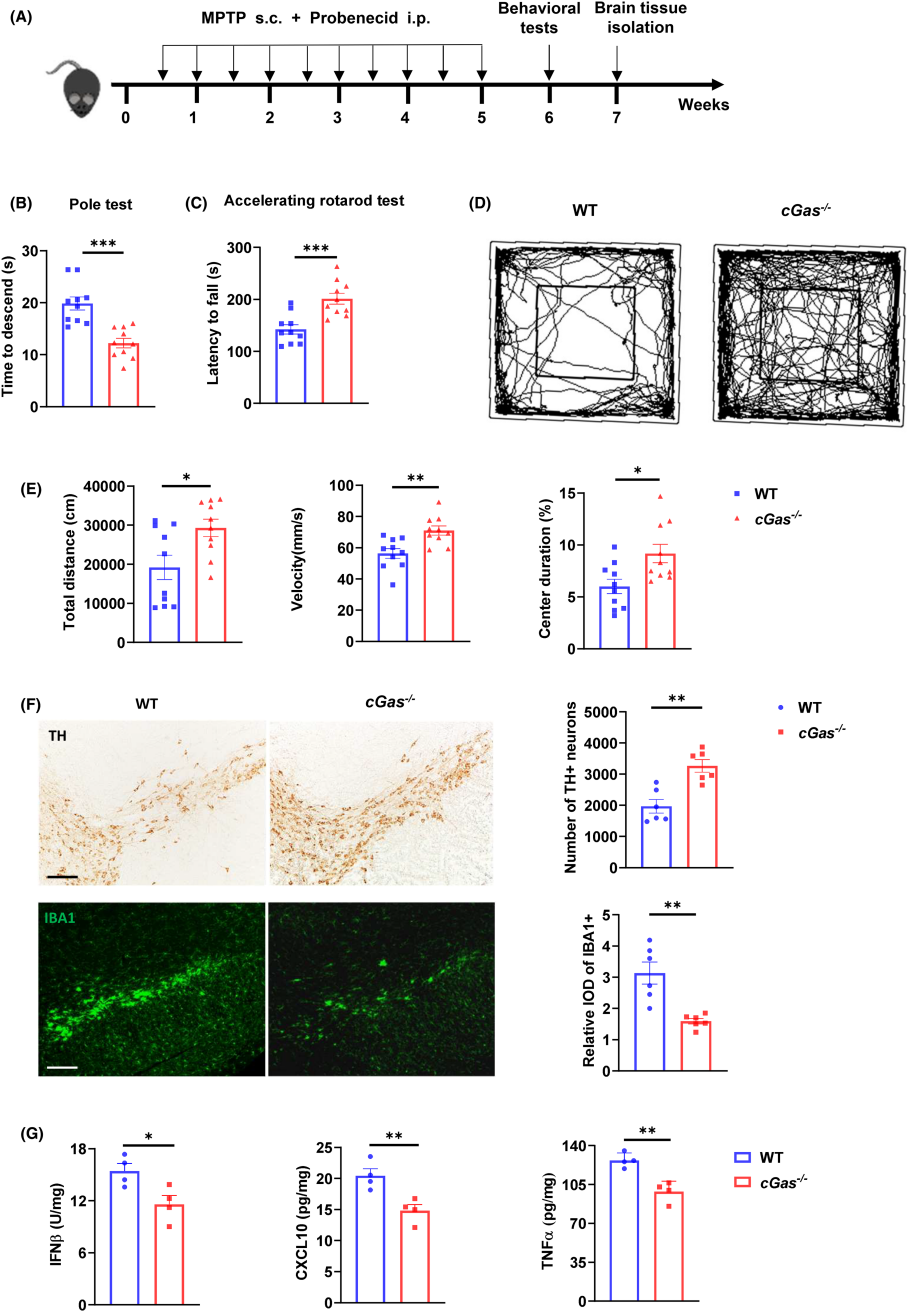
cGAS deficiency protects mice from MPTP/p‐induced toxicity. (A) Schematic representation of the experiments in (B–G). (B) Time to descend during pole test analysis of MPTP‐induced WT or *cGas* ^−/−^ mice (*n* = 10/group). (C) Latency to fall during the accelerating rotarod test analysis of MPTP‐induced WT or *cGas* ^−/−^ mice (*n* = 10/group). (D and E) Open field test analysis of MPTP‐induced WT or *cGas* ^−/−^ mice (*n* = 10/group). Data are presented as representative movement tracks in (D), quantified total traveled distance, movement speed and percentage of center area duration in (E) (*n* = 10/group). (F) Immunohistochemistry and immunofluorescence analysis of TH neurons and microglia (IBA1, green) in the brain SN tissue from indicated mice (*n* = 6/group). Data are presented as representative pictures (left), quantified number of TH neurons and relative IOD of IBA1 (right). Scale bar, 200 μm. (G) ELISA analysis of IFNβ, CXCL10 and TNFα in the brain SN tissue of PBS or MPTP‐induced mice (*n* = 4/group). Data are pooled from three independent experiments. Error bars show means ± SEM. Multiple *t*‐test.

Probenecid can avoid renal clearance of neurotoxins and increase the production of toxic metabolite MPP^+^ level, resulting in a significant irreversible loss of DA neurons and thereby inducing chronic neurotoxic PD. To further confirm that cGAS controls toxicity induced by MPTP, the *cGas* ^−/−^ and WT mice were injected with MPTP and probenecid (MPTP/p) (Figure 3A). *cGas* ^−/−^ mice were more resistant to MPTP/p than WT mice, as indicated by fewer motor deficits on the pole test, accelerating rotarod, and open field test in MPTP/p‐treated *cGas* ^−/−^ mice (Figure 3B–E). Consistent with behavioral tests, the pathological analysis showed less loss of dopamine neurons, decreased microglial activation, and decreased the number of GFAP^+^ astrocytes in SN tissue and dorsal striatum area of *cGas* ^−/−^ mice (Figure 3F, Figure S2A). ELISA and QPCR analysis displayed lower expression in cGAS‐associated pro‐inflammatory factors shown above in SN tissue of *cGas* ^−/−^ mice (Figure 3G, Figure S2B). These results suggest that cGAS protects mice from MPTP/p‐induced neurotoxicity and neuroinflammation.

### 3.3 cGAS deficiency in CNS resident cells controls MPTP‐induced neurotoxicity and neuroinflammation

**FIGURE 4:**
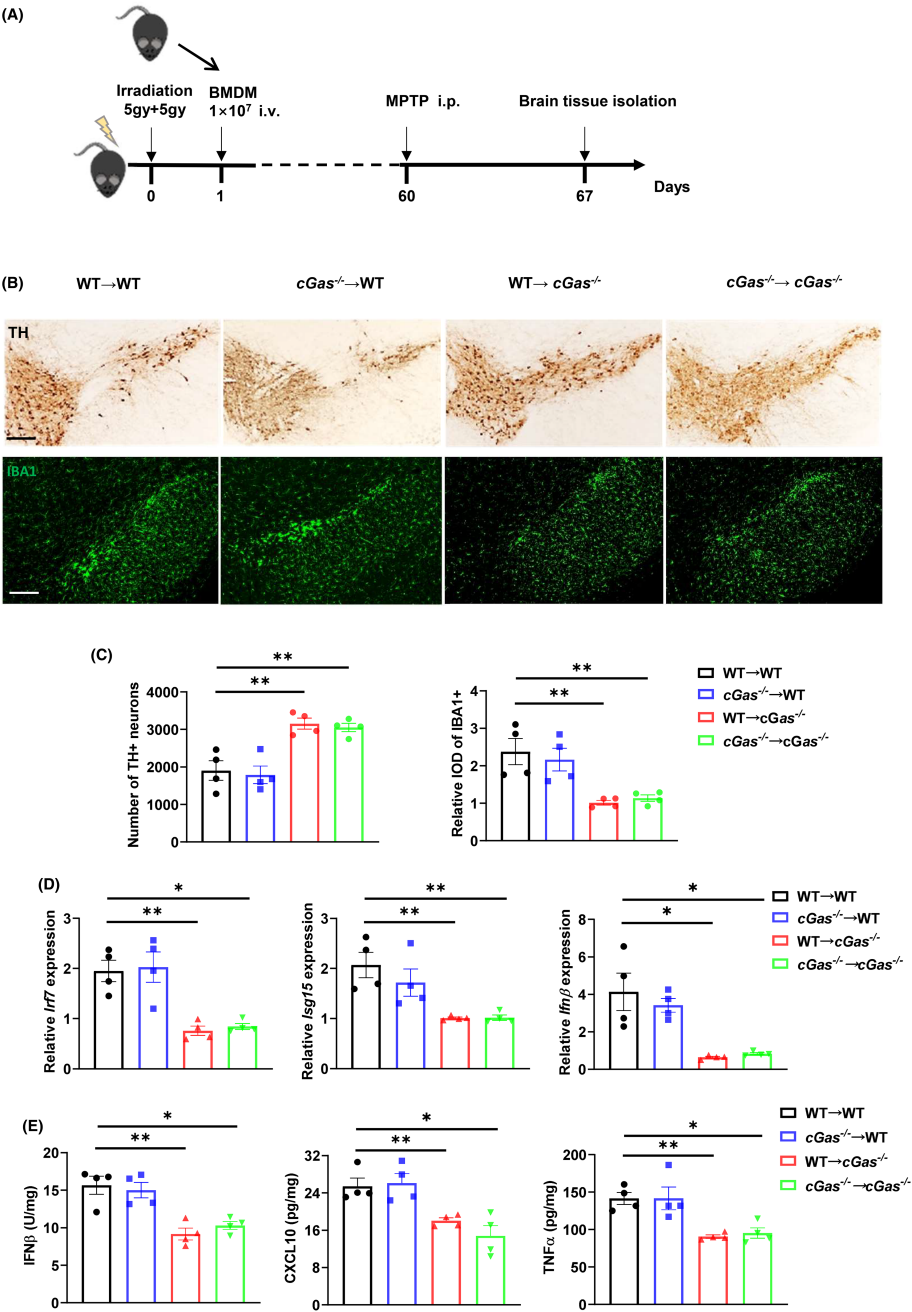
cGAS deficiency in CNS resident cells protects against MPTP toxicity. (A) Schematic representation of the experiments in (B–E). (B and C) Immunohistochemistry and immunofluorescence analysis of TH neurons and microglia (IBA1, green) in the brain SN tissue from indicated mice (*n* = 4/group). Data are presented as representative pictures in (B), quantified number of TH neurons and relative IOD of IBA1 in (C). Scale bar, 200 μm. (D) Quantitative PCR analysis of indicated genes in the brain SN tissue from indicated mice (*n* = 4/group). Data were normalized to the reference gene, *Hprt*. (E) ELISA analysis of IFNβ, CXCL10 and TNFα in the brain SN tissue from indicated mice (*n* = 4/group). Data are pooled from two independent experiments. Error bars show means ± SEM. Multiple *t*‐test.

To further determine whether cGAS deficiency in peripheral immune cells or CNS resident cells contributes to the suppression of MPTP‐induced toxicity, lethally irradiated WT and *cGas* ^−/−^ mice were adoptively transferred with WT or *cGas* ^−/−^ bone marrow cells to reconstitute chimeric mice. Two months after bone marrow transplantation, the four chimeric groups were treated with MPTP (Figure 4A). The *cGas* ^−/−^ recipient mice remained highly resistant to MPTP treatment regardless of their bone marrow transplant donor (WT → *cGas* ^−/−^ or *cGas* ^−/−^ → *cGas* ^−/−^). Notably, the transplant of WT bone marrow did not improve the loss of TH‐positive neurons, decrease the number of activated microglial cells and GFAP^+^ astrocytes, or reduce levels in factors associated with cGAS‐dependent inflammation (Figure 4B–E, Figure S3A–C). This suggests that cGAS in CNS resident cells are responsible for controlling MPTP‐induced neurotoxicity and neuroinflammation. Accordingly, public datasets in GEO showed that *cGAS*, *STING*, and *IFNα1* expression were higher in SNs of PD patients than in healthy donors (Figure 1A).^28^ Reciprocal transfer of bone marrow from *cGas* ^−/−^ mice did not improve disease severity in WT mice (*cGas* ^−/−^ → WT), which were equivalent to WT controls (WT → WT), as indicated by the comparable number of TH‐positive neurons and activated microglia, and levels of cGAS‐dependent inflammatory factors (Figure 4B–E, Figure S3A–C), suggesting that cGAS in peripheral immune cells had no effects on MPTP‐induced neurotoxicity and neuroinflammation. Consistent with our results, public datasets in GEO showed that *cGAS*, *TBK1*, *IRF3*, and *IFNβ1* expression in peripheral blood mononuclear cells (PMBCs) remained comparable between PD patients and healthy donors (Figure S3D).^29^ These results indicate that cGAS deficiency in the CNS resident cells but not peripheral immune cells protect against MPTP‐induced neurotoxicity and neuroinflammation.

### 3.4 cGAS deficiency inhibits microglial antiviral‐related inflammatory gene expression during MPTP treatment

**FIGURE 5:**
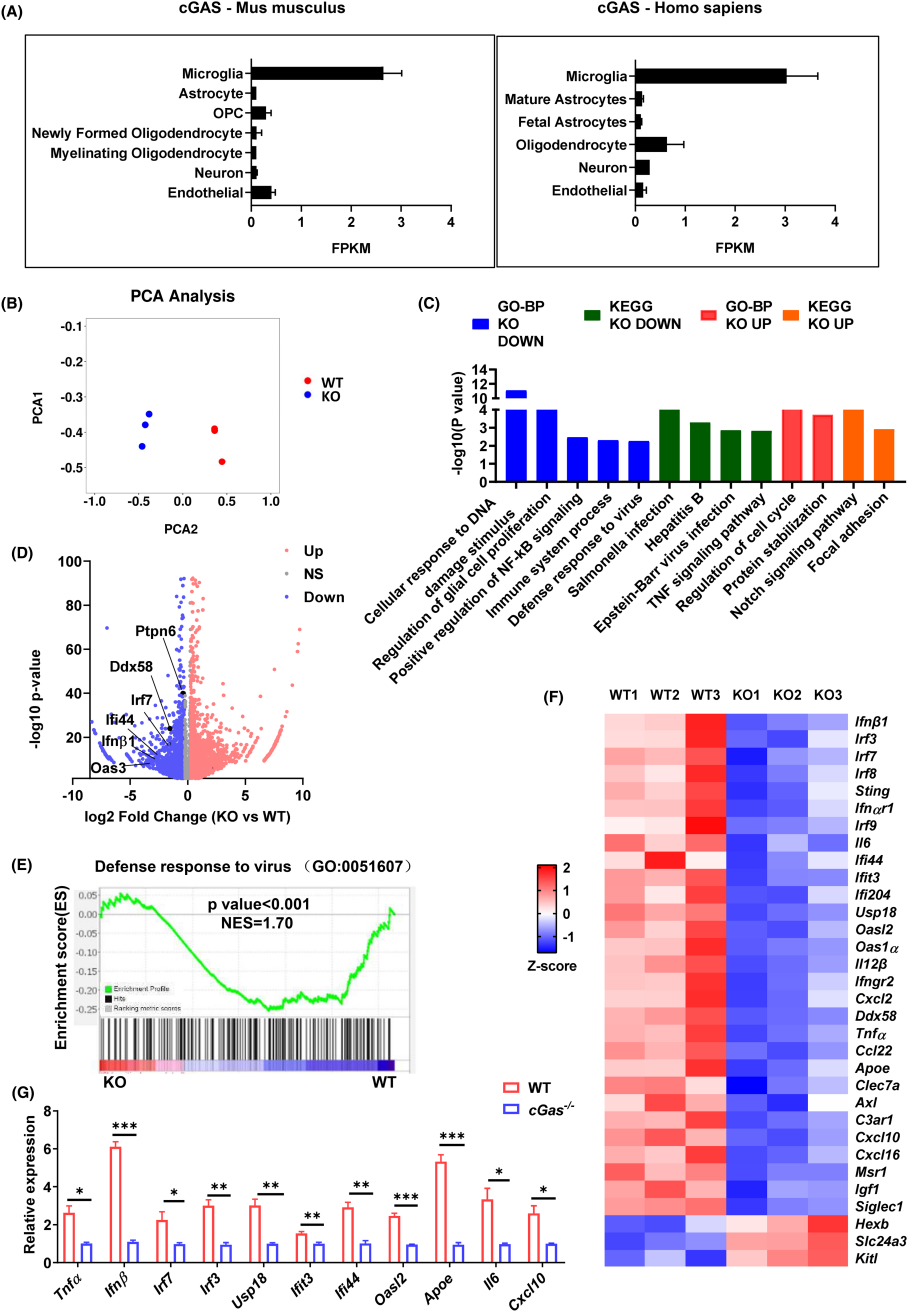
Microglial cGAS enhances the antiviral‐related inflammatory genes expression during MPTP treatment. (A) Mouse and human *cGas* gene expression in different types of CNS cells from public database (https://www.brainrnaseq.org/). (B) WT and *cGas* ^−/−^ mice were treated with MPTP, and then the microglia were sorted to perform RNA‐seq. Principal component analysis showing transcriptional clustered architecture in microglia from WT and *cGas* ^−/−^ microglia. (C) GO‐BP and KEGG pathway analysis identification of the most significantly enriched signaling pathways in the WT and *cGas* ^−/−^ microglia. (D) Volcano plot displaying the results of differential gene expression analysis performed in the WT and *cGas* ^−/−^ microglia. Genes related with ISG signature are indicated. (E) GSEA of the genes associated with the “defense response to virus” in microglia sorted from brain SN tissue of WT and *cGas* ^−/−^ mice in (C) based on the GO‐BP Database. Nominal *p* < 0.001. Normalized Enrichment Score (NES) = 1.70. (F) Heatmap of genes with an adjusted *p*‐value < 0.05, false discovery rate <0.05, and fold‐change >1.1 from RNA‐seq analysis of microglia in (B). (G) Quantitative PCR analysis of indicated genes of microglia from brain SN tissue of WT and *cGas* ^−/−^ mice (*n* = 3/group). Data were normalized to the reference gene, *Hprt*. Error bars show means ± SEM. Multiple *t*‐test.

A public database showed that cGAS was highly expressed in microglia compared to other CNS cell types in mice and humans, such as astrocytes, oligodendrocytes (OPC), neurons, and endothelial cells (Figure 5A). Consistently, cGAS has been reported to be expressed mainly in microglia in CNS.^30^ Therefore, we next focused on exploring microglial cGAS. To further dissect the mechanistic role of microglial cGAS in exacerbating MPTP‐induced neurotoxicity and neuroinflammation, we performed RNA‐seq using microglia isolated from SN of WT and *cGas* ^−/−^ mice on day 7 after MPTP treatment (Figure S4A). Principal component (PC) analysis showed that WT microglia had a distinct gene‐clustered architecture compared with the communities of *cGas* ^−/−^ microglia (Figure 5B). Gene ontology biological process (GO‐BP) analysis showed that the top biological processes downregulated in *cGas* ^−/−^ microglia were related to DNA damage stimulated cellular responses (Figure 5C). Kyoto Encyclopedia of Genes and Genomes (KEGG) further confirmed the top down‐regulated pathway in *cGas* ^−/−^ microglia was Salmonella infection, Hepatitis B virus infection, and Epstein–Barr virus infection (Figure 5C). Interestingly, volcano plot analysis displayed significantly decreased expression in many genes related to ISG signature, such as *Ptpn6*, *Ddx58*, *Irf7*, *Ifnβ1*, *Ifi44*, and *Oas3* in *cGas* ^−/−^ microglia (Figure 5D). Gene set enrichment analysis (GSEA) confirmed a downregulation of genes involved in the virus defense response in *cGas* ^−/−^ mice (Figure 5E). Heatmap and qPCR analysis also showed significantly decreased expression of genes associated with many antiviral pathways and damage DNA sensing pathways, such as *Tnfα*, *Irf3*, *Irf7*, *Ifnβ1*, *Ifit3*, *Oasl2*, *Il6*, and *Cxcl10* (Figure 5F,G). Interestingly, the expression of a disease‐associated microglia (DAM) signature, such as *Apoe*, *Clea7c*, and *Axl*, was significantly decreased in *cGas* ^−/−^ microglia (Figure 5F,G). These results suggest that cGAS deficiency restricts the microglial antiviral pathway, facilitating microglial inflammation during MPTP treatment.

### 3.5 cGAS deficiency inhibits neuronal death and controls the phenotypic switch of microglia and astrocytes from a homeostatic to an inflammatory state

**FIGURE 6:**
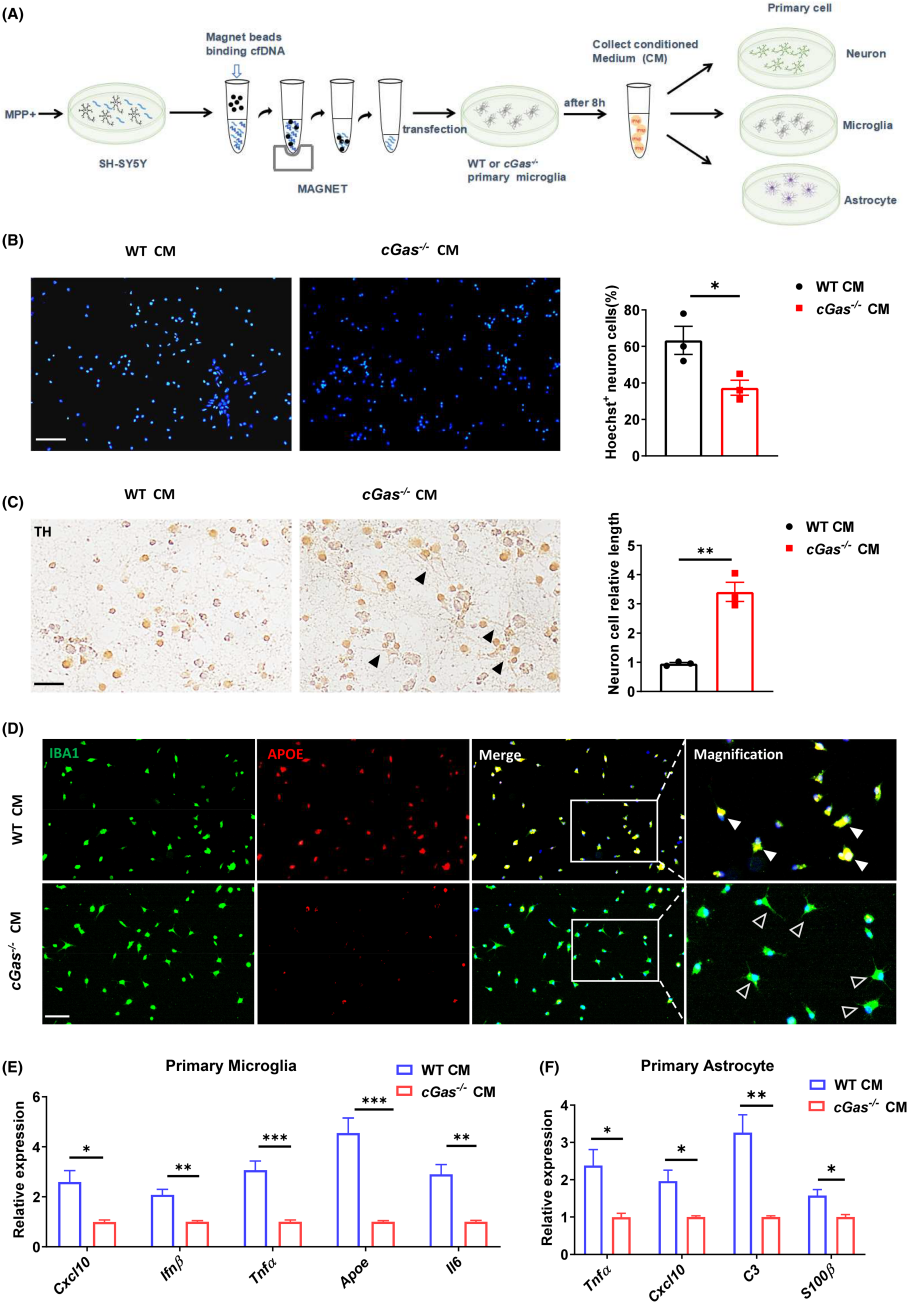
cGAS promotes neurons death, and drives inflammatory phenotypic switch of microglia and astrocytes in vitro. (A) Schematic representation of the experiments in (B–F). Briefly, cell‐free dsDNA in cell culture medium released from the MPP^+^ (5 mM) treated SH‐SY5Y neuronal cell line was collected and transfected into primary WT or *cGas* ^−/−^ microglia. After 8 h, CM was collected to culture primary neurons, microglia, and astrocytes for 24 h. (B and C) Hoechst staining and Immunohistochemistry analysis of primary neurons treated with CM from WT or *cGas* ^−/−^ microglia for 24 h. Black solid arrowheads indicate the TH‐positive neuron axons. Scale bars, 50 μm. (D) Immunofluorescence analysis of APOE (red) expression in the primary microglia treated with CM from WT or *cGas* ^−/−^ microglia for 24 h. White hollow arrowheads indicate the branched protrusions of microglia. The merge of IBA1 with APOE is indicated by solid white arrowheads. Scale bars, 50 μm. (E) Quantitative PCR analysis of indicated genes in the primary microglia from (D). Data were normalized to the reference gene, *Hprt*. (F) Quantitative PCR analysis of indicated genes in the primary astrocytes treated with CM from WT or *cGas* ^−/−^ microglia for 24 h. Data were normalized to the reference gene, *Hprt*. Data are representative of three independent experiments. Error bars show means ± SEM. Unpaired *t*‐test for (B) and (C). Multiple *t*‐test for (E) and (F).

The dramatic loss of dopaminergic neurons occurs in the SN during PD. Indeed, we observed a significant increase of γH2AX, the marker of DNA damage, in the SN of mice treated with MPTP (Figure S5A). We next determined whether cGAS‐dependent neuroinflammation and neurotoxicity in the MPTP‐induced PD mice were caused by microglial activation. We firstly detected decreased levels of IFNβ in conditioned medium (CM) from *cGas* ^−/−^ microglia transfected with dsDNA from damaged neurons compared with that of WT, suggesting cGAS‐STING was indeed activated by cf‐dsDNA from damaged neurons (Figure S5B). We then measured the effects of CM from *cGas* ^−/−^ and WT microglia on neuronal survival and phenotypic switch of microglia and astrocytes (Figure 6A). The CM from *cGas* ^−/−^ microglia enhanced the survival of neurons and restricted neuronal axon length shortening compared with that of WT (Figure 6B,C). In addition, the CM from *cGas* ^−/−^ microglia strongly inhibited the expression of DAM‐associated pro‐inflammatory genes, such as *Cxcl10*, *Ifnβ*, *Tnfα*, *Apoe*, and *Il6* in the primary microglia compared with that of WT (Figure 6D,E). Consistently, the CM from *cGas* ^−/−^ microglia also significantly inhibited the expression of neurotoxic A1‐related factors, such as *Tnfα*, *Cxcl10*, and *C3*, but elevated the expression of neuroprotective A2‐related genes, such as *S100β*, in the primary astrocytes compared with that of WT (Figure 6F, Figure S5C). These data suggest that microglial cGAS mediates the phenotypic switch of microglia and astrocytes from a homeostatic to an inflammatory state and neuronal death in vitro.

### 3.6 The administration of cGAS inhibitors attenuates the neurotoxicity and neuroinflammation

**FIGURE 7:**
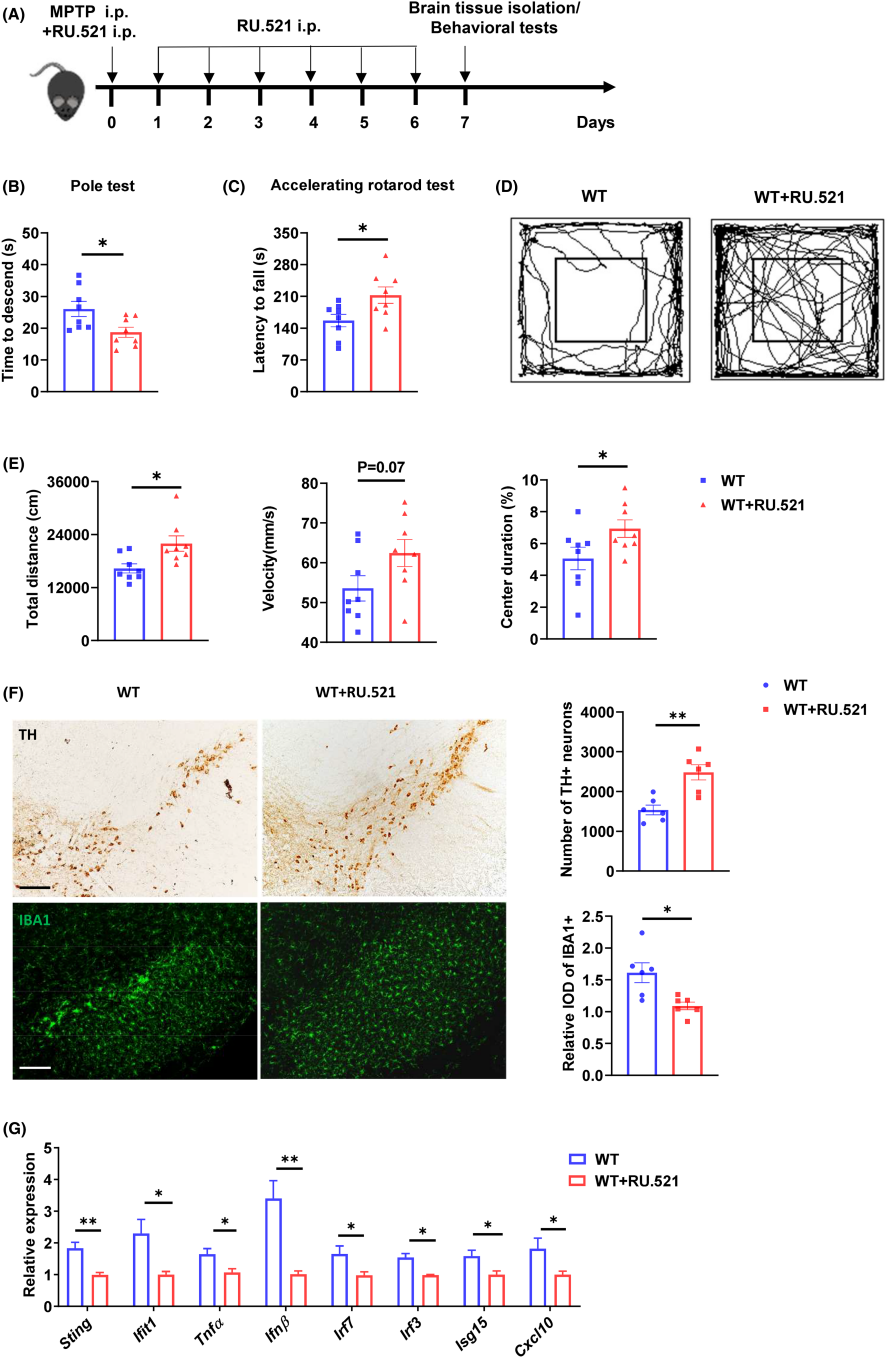
The administration of cGAS inhibitors restricts MPTP toxicity. (A) Schematic representation of the experiments in (B–G). (B) Time to descend during pole test analysis of MPTP‐treated WT mice (*n* = 8/group). (C) Latency to fall during the accelerating rotarod test analysis of MPTP‐treated WT mice (*n* = 8/group). (D and E) Open field test analysis of MPTP‐treated WT mice (*n* = 8/group). Data are presented as representative movement tracks in (D), quantified total traveled distance, movement speed and percentage of center area duration in (E) (*n* = 8/group). (F) Immunohistochemistry and immunofluorescence analysis of TH neurons and microglia (IBA1, green) in the brain SN tissue from indicated mice (*n* = 6/group). Data are presented as representative pictures (left), quantified number of TH neurons and relative IOD of IBA1 (right). Scale bar, 200 μm. (G) Quantitative PCR analysis of indicated genes in the brain SN tissue from indicated mice (*n* = 5/group). Data were normalized to the reference gene, *Hprt*. Data are pooled from two independent experiments. Error bars show means ± SEM. Multiple *t*‐test.

To confirm that cGAS restricts MPTP‐induced neurotoxicity and neuroinflammation, we treated WT mice intraperitoneally with the cGAS inhibitor RU.521 (Figure 7A). We found that RU.521 attenuated PD‐related behavior phenotypes, reduced the loss of TH‐positive neurons, decreased the number of activated microglial cells in the SN, and lowered levels in factors associated with cGAS‐dependent inflammation (Figure 7B–G). These results suggested that blocking cGAS can be an efficient therapeutic strategy for PD.

## 4 DISCUSSION

It has been reported that DNA damage affects mechanisms central to PD pathogenesis, including protein homeostasis, mitochondrial function, and redox homeostasis.^31^, ^32^ Therefore, DNA damage strongly implies the capacity to induce activation of the cGAS‐STING pathway. Similarly, we show that cGAS‐STING pathway activation contributes to neuronal damage and neuroinflammation during MPTP treatment.

Over the years, many studies have tried to understand the roles of neuroinflammation in driving PD and other neurodegenerative diseases. A variety of studies showed that, except for AIM2, several PRRs, such as NLRP3, TLR2, TLR4, and TLR9, exacerbate PD progression by promoting neuroinflammation in microglia.^33^, ^34^, ^35^ Consistently, we found that cGAS enhances the disease severity of PD, by aggregating PD‐related behavior, and decreasing the loss of TH‐positive neurons and the number of activated microglial cells in *cGas* ^−/−^ mice during PD. While it has been widely considered that peripheral macrophages are implicated in the pathogenesis of PD,^36^, ^37^, ^38^ we identified that cGAS in CNS resident cells but not peripheral immune cells accelerate disease progression of PD by bone marrow chimeric experiments. We further showed that microglial cGAS contribute to PD development by analyzing cGAS expression in CNS cells but without further evidence from conditional knockout mice. Notably, consistent with our findings, a recent study reported that STING‐deficient mice resist PD induction using αSyn‐preformed fibril.^39^ Still, the role of cGAS in PD progression and the efficacy of targeting the cGAS‐STING pathway in PD treatment in vivo were unknown. Here, by using knockout mice and a cGAS inhibitor in the MPTP‐induced PD mouse models, we demonstrate that cGAS ablation mediates neuroprotection against MPTP.

We performed RNA‐seq analysis of microglia in SN from WT and *cGas* ^−/−^ mice treated with MPTP. We revealed that the most strongly downregulated responses in *cGas* ^−/−^ microglia were associated with the antiviral pathway. Previous studies have shown that the increased levels of type I interferon (IFN‐I), the antiviral cytokine, are correlated with senescence and aging.^40^, ^41^ The inflammatory microenvironment of microglia has been reported to shape the microglial phenotype switch between homeostasis and DAM.^42^ Moreover, our in‐vitro data revealed that cGAS restricts excessive inflammatory responses in microglia and astrocytes and neuronal death by attenuating microglial antiviral pathway signaling.

Historically, neuroinflammation has been widely considered a cause of neurodegeneration and not an effect^43^; recent efforts in therapeutic intervention seek to prevent neuroinflammation in PD. Most proposed inflammation‐modulating therapies target non‐specifically, thus resulting in a broad range of side effects.^44^ Recently, exploring TLRs in PD provides a specific route for therapeutic development. Notably, NF‐κB, the major pro‐inflammatory transcription factor of TLRs pathways, can be targeted for an effective treatment for PD.^45^ It is well established that Mucuna pruriens, ursolic acid and chlorogenic acid exhibit anti‐inflammatory and neuroprotective activity by inhibiting NF‐κB in the MPTP‐induced PD mouse model.^45^, ^46^ However, targeting TLRs attenuates neuroinflammation mainly triggered by aggregated α‐synuclein and gut dysbiosis.^33^ In our study, cGAS inhibitor RU.521 significantly attenuates the toxicity of MPTP by suppressing antiviral‐associated neuroinflammation. Therefore, targeting the cGAS‐STING signaling pathway shows potential as a therapeutic strategy for treating virus or damaged DNA fragment‐induced PD pathogenesis.

The two main experimental animal models for PD are the genetic and toxin models. The transgenic models only simulate the familial form of PD, which accounts for only 10% of PD subjects.^47^ The standard toxin models, to some extent, phenocopy the salient feature of PD, especially in sporadic PD, which accounts for an overwhelming majority of PD subjects.^47^, ^48^ Although the MPTP‐induced mouse toxin model does not fully recapitulate motor deficits of PD patients, it is considered the gold standard in PD research, due to its low cost and significant clinical correlation over other models.^48^, ^49^, ^50^ Moreover, 1‐methyl‐4‐phenylpyridinum (MPP^+^), the final toxic metabolite of MPTP, has been reported to trigger an inflammatory process characterized by microglia activation in the SN and striatum^51^; thus, the MPTP‐induced mouse model is suitable for studying the mechanism of neuroinflammation during PD development.

Overall, these findings demonstrate cGAS in microglia driving neuroinflammation in mouse models of PD, suggesting that cGAS is involved in PD progression. Moreover, the neuroinflammatory and neurodegenerative effects of the cGAS‐STING pathway may not be limited to PD, as evidenced by cGAS‐STING activation in other neurodegenerative diseases such as Huntington's disease, Niemann–Pick disease type C, ALS, and AD.^18^, ^52^, ^53^, ^54^ Therefore, targeting the cGAS‐STING signaling pathway highlights the potential for broad‐therapeutic treatment of antiviral‐induced neuroinflammation in PD and other neurodegenerative diseases.

## 5 CONCLUSIONS

In summary, our study demonstrates that cGAS‐STING is activated by damaged dsDNA released from dying dopaminergic neurons during MPTP treatment. cGAS deficiency in microglia, but not peripheral myeloid cells, controlled neuroinflammation and neurodegeneration during PD. Mechanistically, microglial cGAS ablation alleviated the neuronal dysfunction and inflammatory response in astrocytes and microglia by inhibiting antiviral inflammatory signaling. The administration of cGAS inhibitors conferred the mice neuroprotection during MPTP exposure (Figure S6). These results suggested that targeting cGAS may be an efficient therapeutic strategy for PD patients.

## AUTHOR CONTRIBUTIONS

BW, CM, SY, and YL designed and performed the experiments, analyzed the data, and prepared the figures; CM, and BW provided the key technique mentoring, data analysis, and research resources; BW, and SY supervised the project; BW, CM, SY, and YL wrote the manuscript. All authors read and approved the final manuscript.

## FUNDING INFORMATION

This work was supported by the National Key R&D Program of China, (2022YFA1303900 to SY and CM), the National Natural Science Foundation of China (82270539 and 81901227 to CM, 82070567 and 32270921 to SY, 81991523 to BW, 82104146 to SL), the Open Project of State Key Laboratory of Reproductive Medicine of Nanjing Medical University (grant SKLRM‐2021B3 to SY), Self‐selected topic Project of State Key Laboratory of Reproductive Medicine of Nanjing Medical University (grant SKLRM‐2022BP3 to SY), the Open Project of Chinese Materia Medica First‐Class Discipline of Nanjing University of Chinese Medicine (2020YLXK017 to BW), the Natural Science Foundation of Jiangsu Province (BK20221352 to BW), the Priority Academic Program Development of Jiangsu Higher Education Institutions (to BW), the Natural Science Excellent Youth Foundation of Jiangsu Province (SBK2022030080 to CM).

## CONFLICT OF INTEREST STATEMENT

The authors declare no conflicts of interest.

## Supporting information


Figures S1–S6

## Data Availability

All are included in the article and the supplementary data. Raw data and processed files of RNA‐seq have been deposited in the NCBI Sequence Read Archive (SRA) database under the accession code PRJNA904829.
