# Supplementary figures and images for: Microglial cGAS drives neuroinflammation in the MPTP mouse models of Parkinson's disease
Source: CNS Neurosci Ther. 2023 Mar 13;29(7):2018–35. doi: 10.1111/cns.14157 (PMC10324349; doi:10.1111/cns.14157)

S-Fig1

A

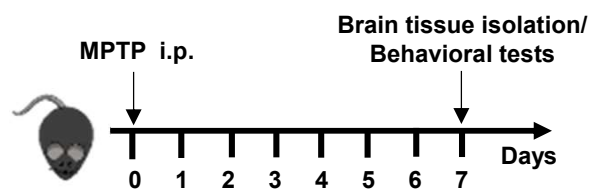

B

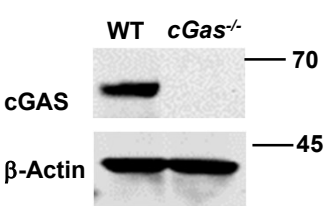

C

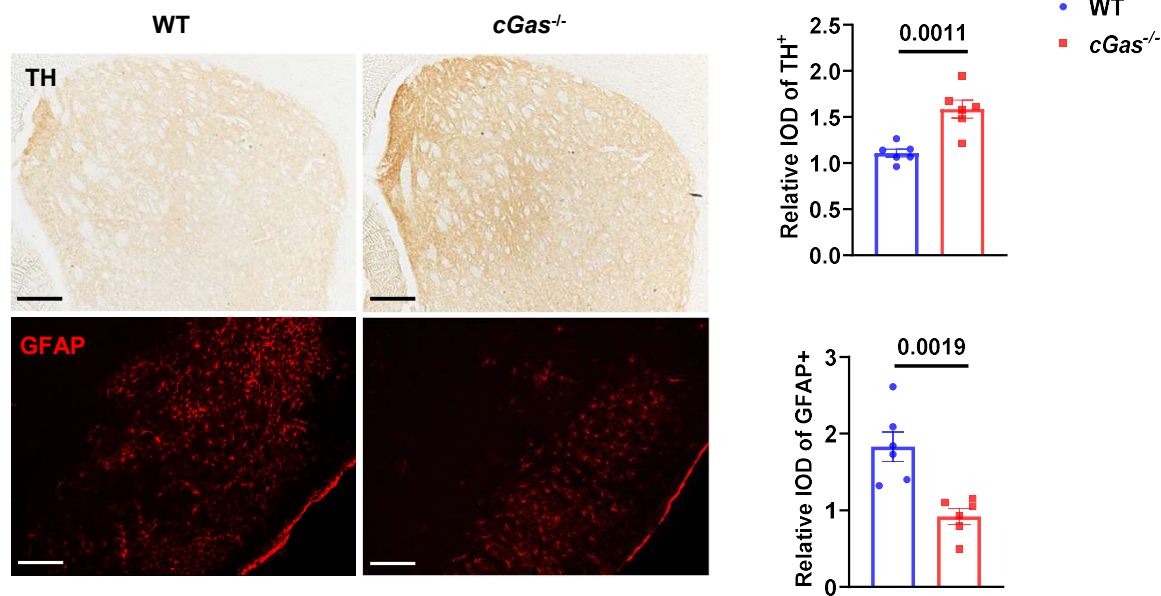

## S-Fig2

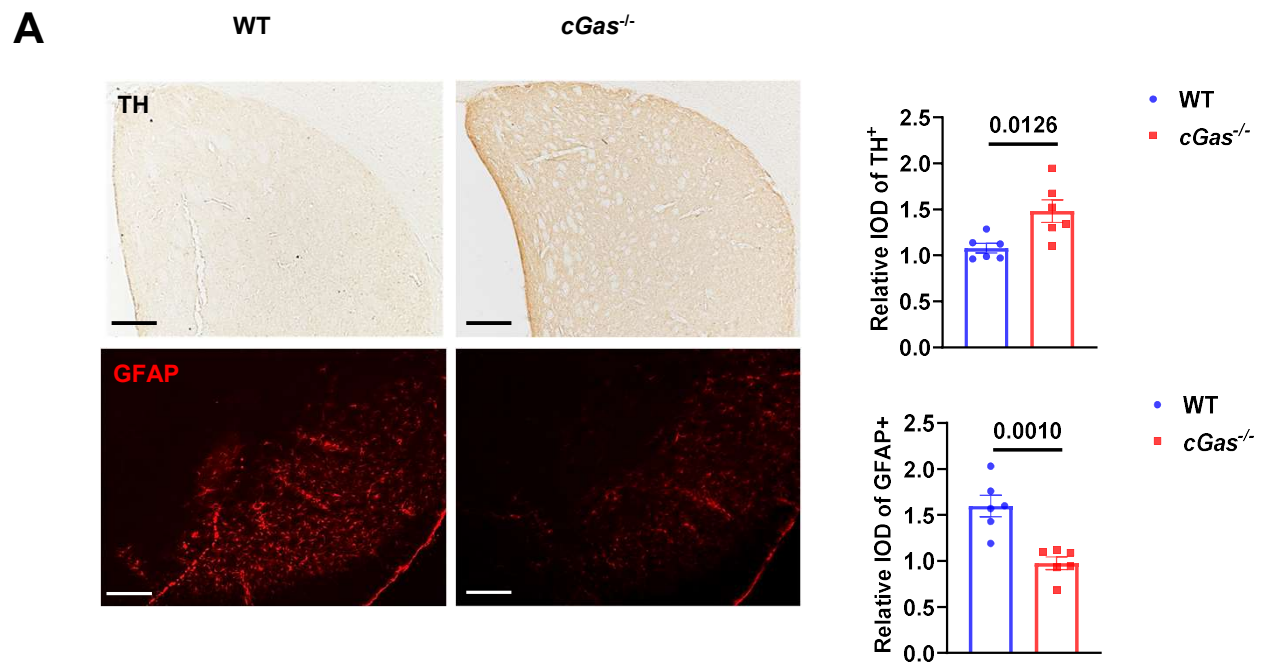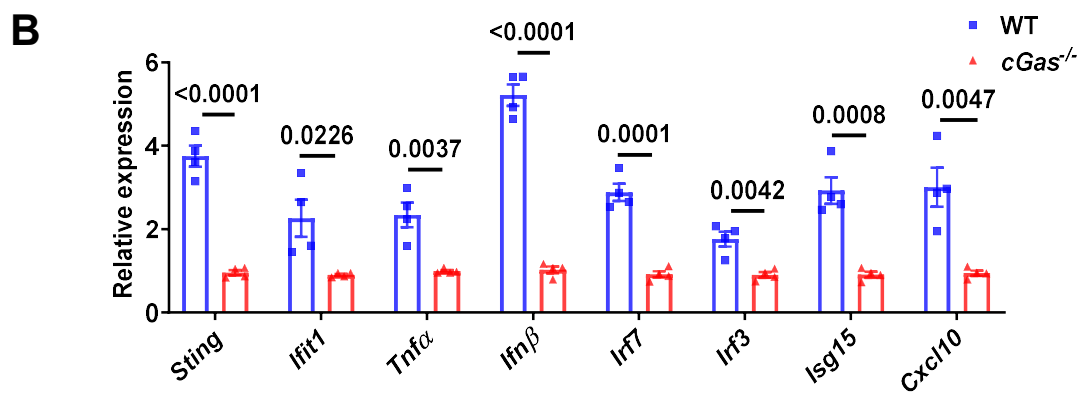

S-Fig3

A

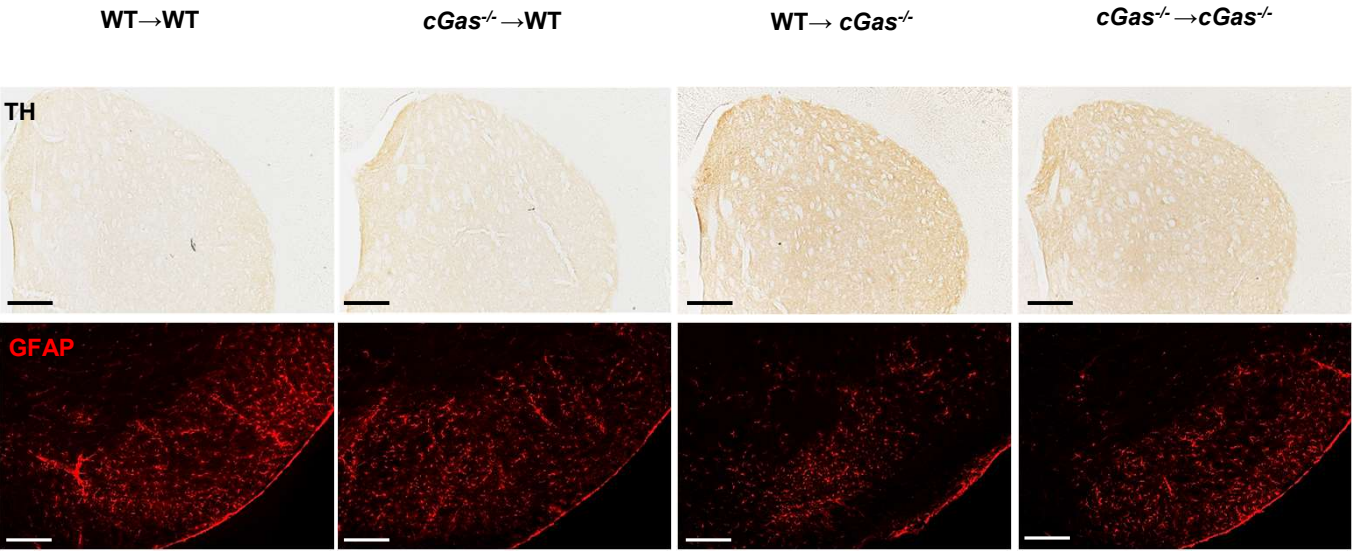

B

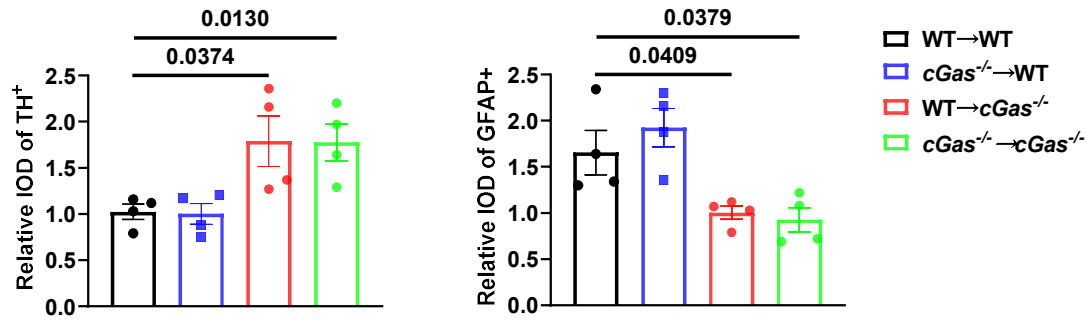

C

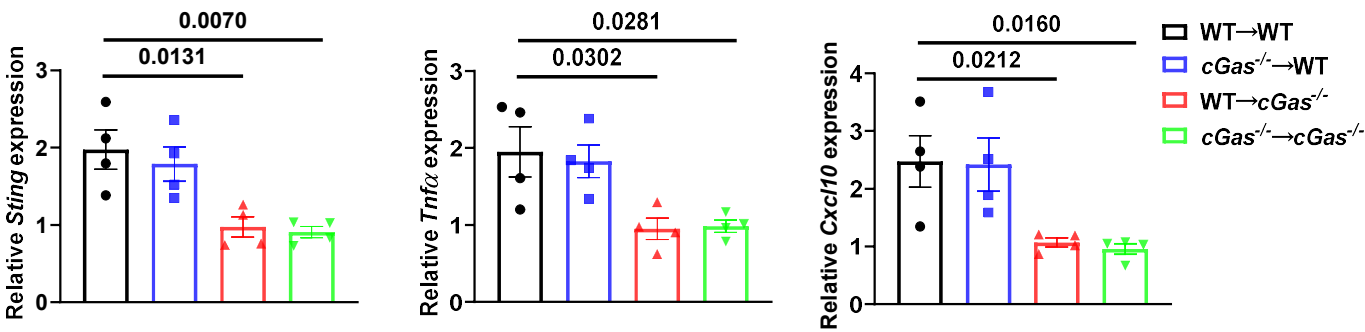

D

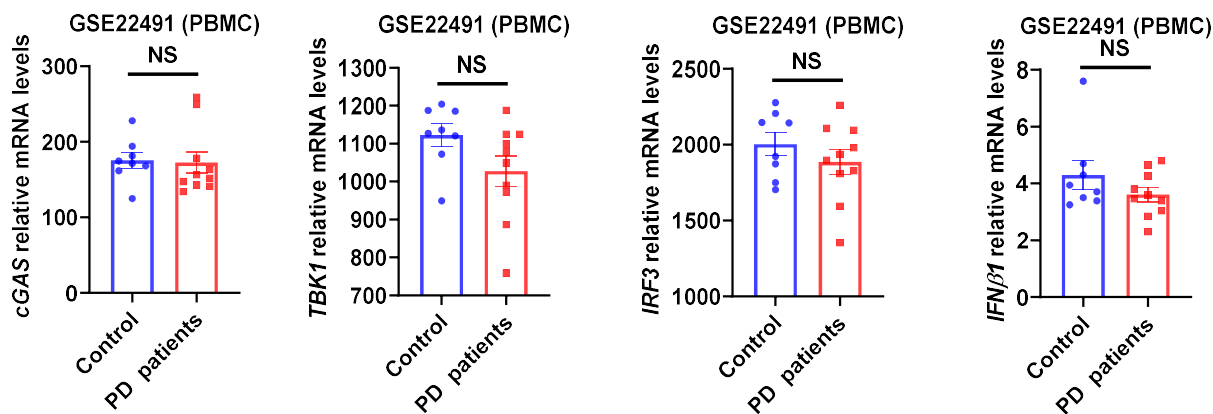

S-Fig4

A

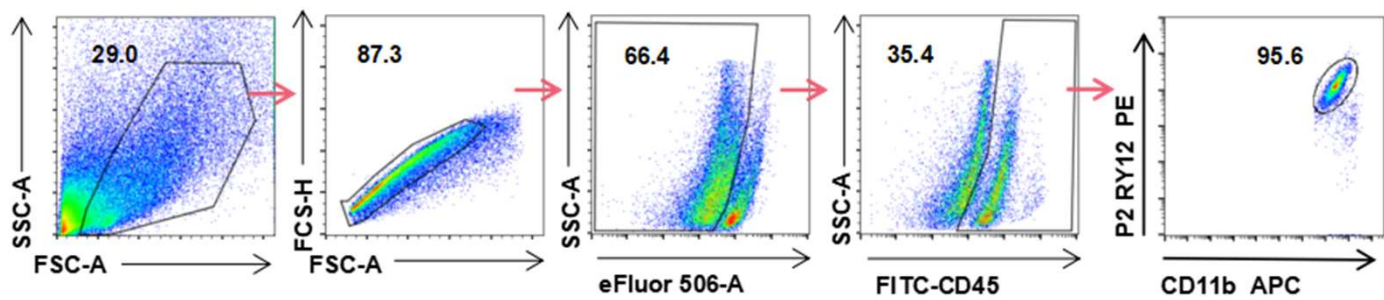

B

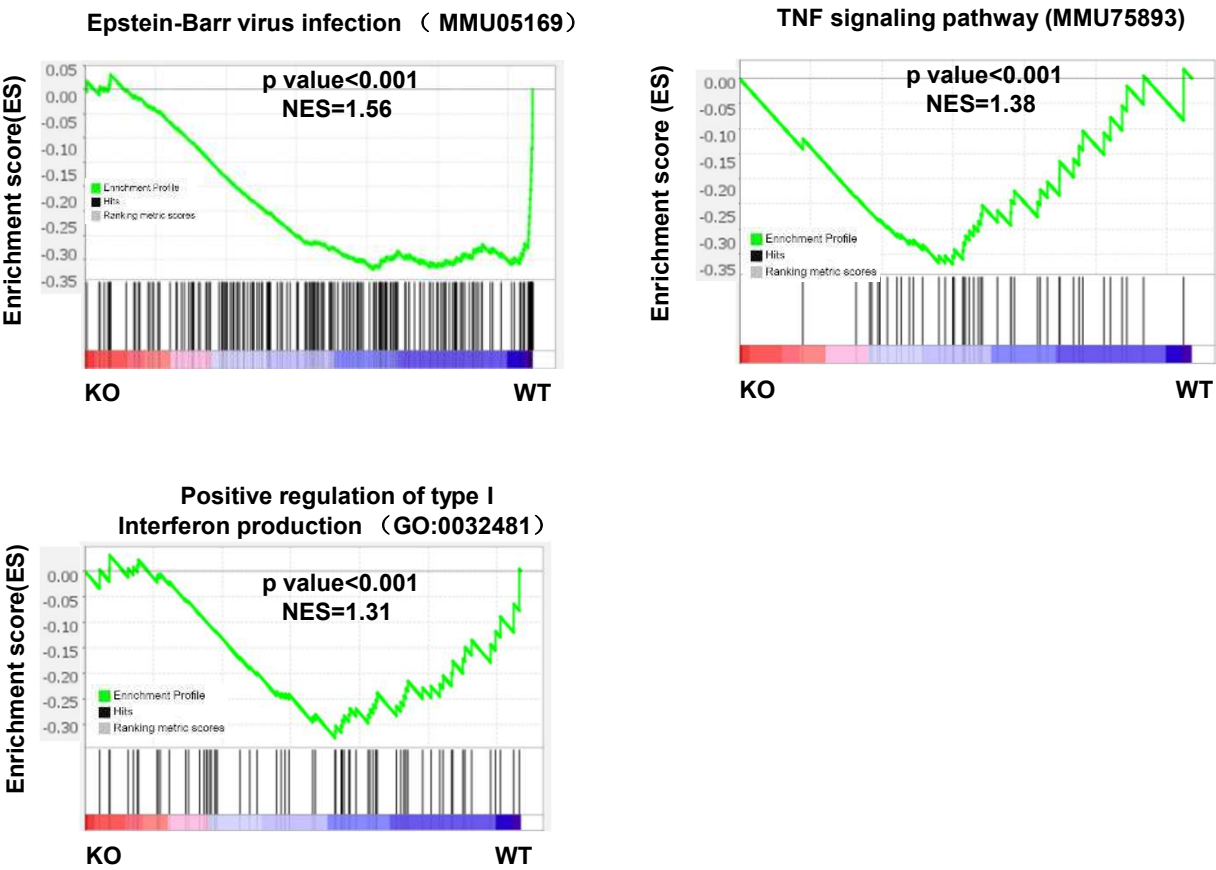

# S-Fig5

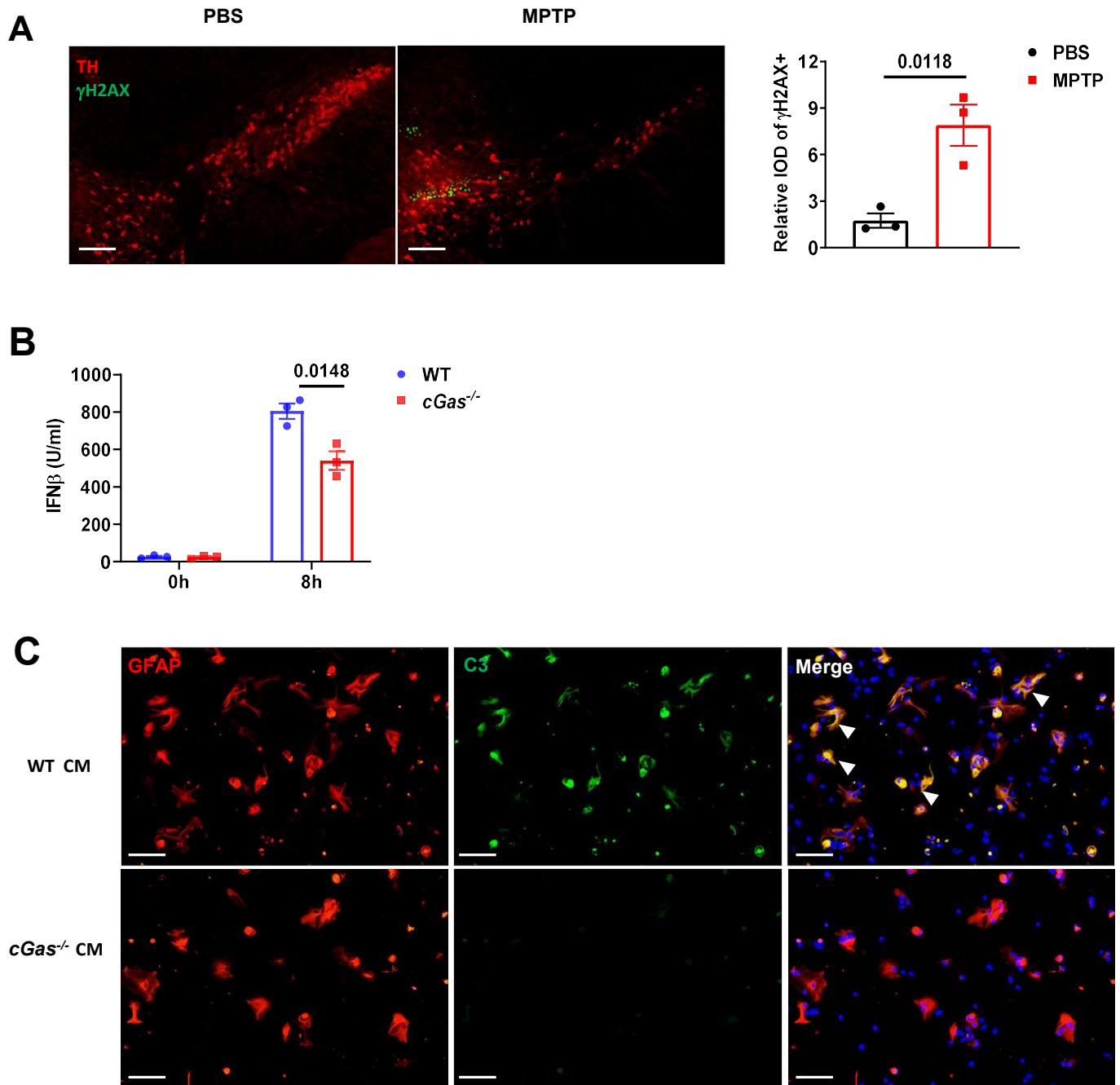

S-Fig6

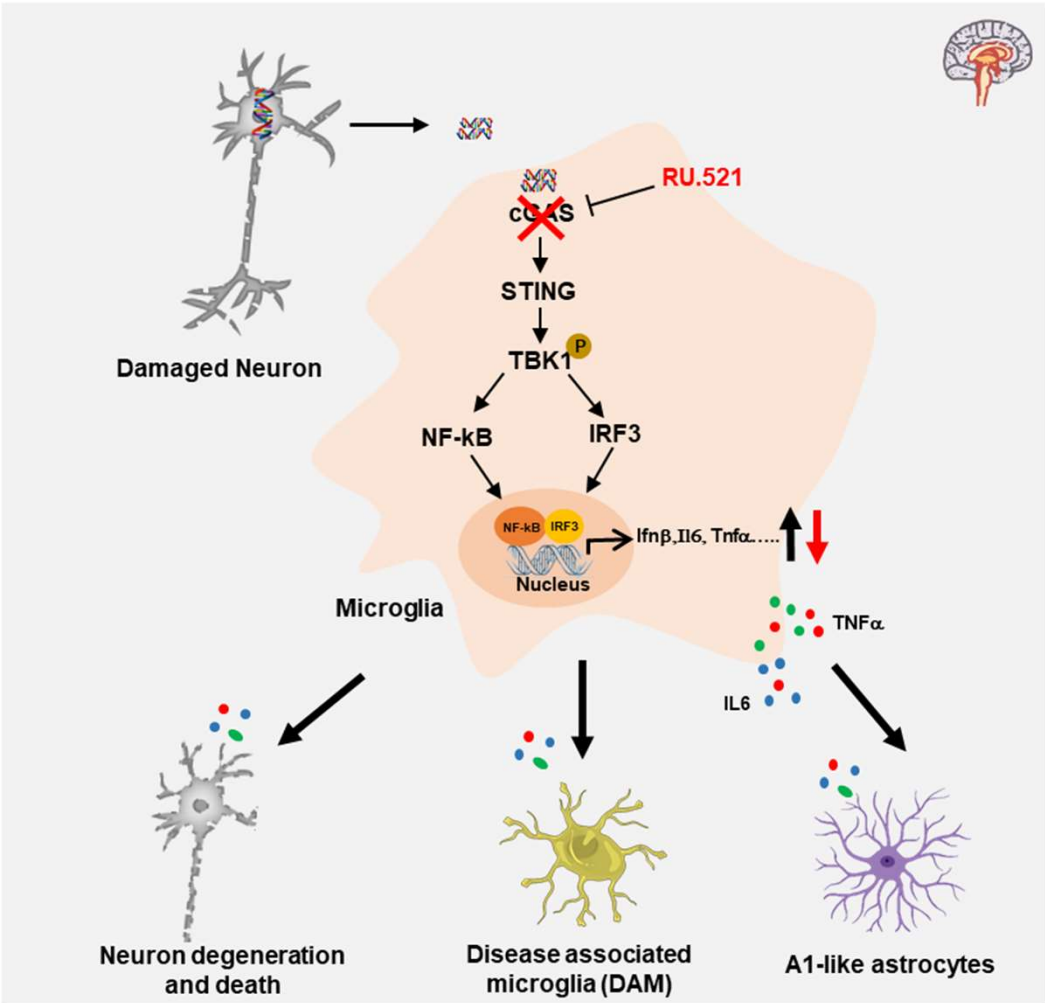

Supplement: Supplementary file 1 — Figures S1–S6 [file CNS-29-2018-s001.zip › CNS_14157_Figures resumission.pdf]

Full unedited gel  
for Figure 1B

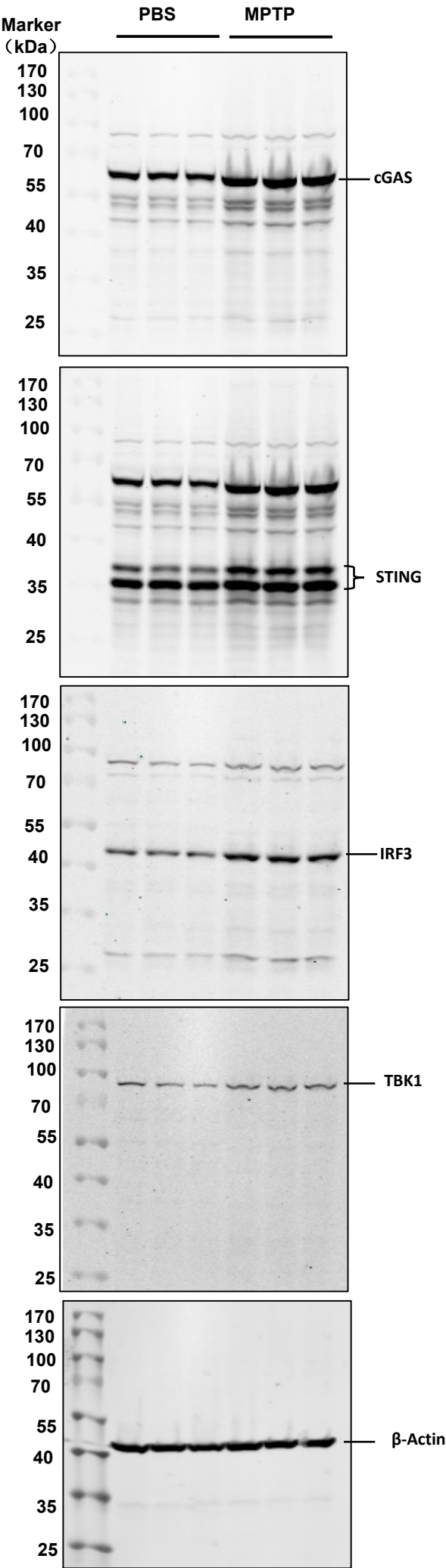

Full unedited gel  
for Figure S1B

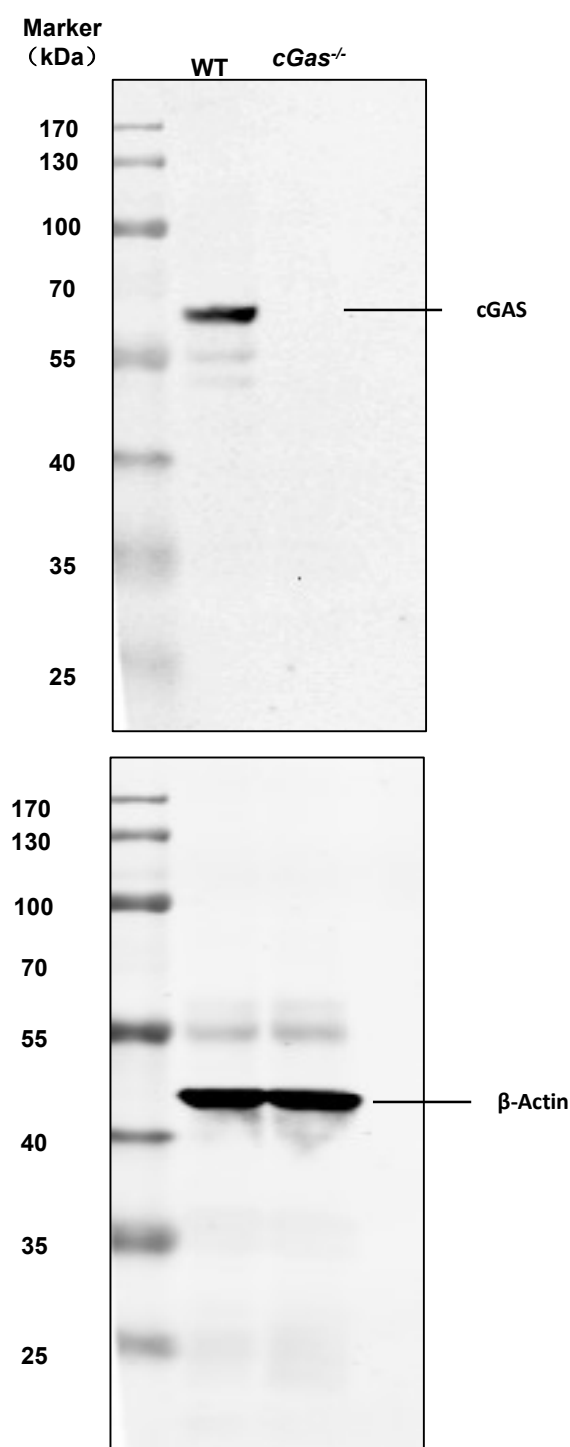

Supplement: Supplementary file 1 — Figures S1–S6 [file CNS-29-2018-s001.zip › CNS_14157_Supplemental Files.pdf]
